# Design Methodology for a Backrest-Lifting Nursing Bed Based on Dual-Channel Behavior–Emotion Data Fusion and Biomechanical Simulation: A Human-Centered and Data-Driven Optimization Approach

**DOI:** 10.3390/biomimetics10110764

**Published:** 2025-11-12

**Authors:** Xiaochan Wang, Cheolhee Cho, Peng Zhang, Shuyuan Ge, Liyun Wang

**Affiliations:** 1 Department of Design, Kyungpook National University, Daegu 41566, Republic of Korea; daegu8599@knu.ac.kr (X.W.); chery@knu.ac.kr (C.C.); 2 School of Arts and Design, Yanshan University, Haigang District, Qinhuangdao 066000, China; 2021327350@knu.ac.kr; 3 Department of Electrical and Electronic Engineering, Kyungpook National University, Daegu 41566, Republic of Korea; geshuyuan@agora.io

**Keywords:** back lifting nursing bed, emotional design, dual-channel data fusion, fuzzy kano model, biomechanical simulation, anybody modeling system, human-machine interaction

## Abstract

Population aging and rising rehabilitation demands highlight the need for advanced assistive devices to improve mobility in individuals with motor impairments. Existing back-support lifting nursing beds often lack adequate human–machine adaptability, safety, and emotional consideration. This study presents a human-centered, data-driven optimization pipeline that integrates behavior–emotion dual recognition, simulation verification, and parameter optimization with user demand mining, biomechanical simulation, and sustainable practices. The design utilizes GreenAI, focusing on low-power algorithms and eco-friendly materials, ensuring energy-efficient AI models and reducing the environmental footprint. A dual-channel data fusion method was developed, combining movement parameters from sit-to-lie transitions with emotional needs extracted from e-commerce reviews using the Term Frequency-Inverse Document Frequency (TF-IDF) and Latent Dirichlet Allocation (LDA) models. The fuzzy Kano model prioritized design objectives, identifying multi-position adjustment, joint protection, armrest optimization, and interaction comfort as key targets. An AnyBody-based human–device model quantified muscle (erector spinae, rectus abdominis, trapezius) and hip joint loads during posture changes. Simulations verified the design’s ability to improve load distribution, reduce joint stress, and enhance comfort. The optimized nursing bed demonstrated improved adaptability across user profiles while maintaining functional reliability. This framework offers a scalable paradigm for intelligent rehabilitation equipment design, with potential extension toward AI-driven adaptive control and clinical validation. This sustainable methodology ensures that the device not only meets rehabilitation goals but also contributes to a more environmentally responsible healthcare solution, aligning with global sustainability efforts.

## 1. Introduction

Movement disorders, characterized by bradykinesia, involuntary movements, and postural instability, are frequently observed in neurological conditions such as stroke and cerebellar injury [1,2,3,4,5]. Stroke remains the leading cause of disability in China, with over 75% of survivors experiencing varying degrees of motor impairment and approximately 40% suffering from severe disability [6,7,8]. For these patients, prolonged bed rest often leads to complications such as pressure ulcers and infections [9,10], placing substantial strain on caregivers and healthcare systems [11]. With the acceleration of population aging, the demand for rehabilitation equipment with enhanced performance, safety, and intelligent functionality continues to grow [12,13].

Despite this growing need, existing rehabilitation and nursing devices often exhibit poor ergonomic adaptability, limited functionality, and suboptimal human–machine interaction, making them insufficient to address the personalized rehabilitation and emotional care requirements of individuals with movement disorders, including those with disorders of consciousness or neurodegenerative syndromes such as Parkinson’s or Alzheimer’s disease [14,15]. Our approach is not only aimed at improving functionality but also considers emotional and psychological needs, thus expanding the device’s potential impact on patients beyond just physical rehabilitation. The backrest-lifting nursing bed, which supports patient transfers and facilitates transitions between sitting and lying postures, has gained widespread application in clinical, eldercare, and home rehabilitation settings [16,17]. However, current designs require urgent functional upgrades to improve user comfort, safety, and adaptability [18]. Advances in ergonomics, biomechanics, and healthcare policy have promoted the development of devices with multi-degree-of-freedom adjustments, intelligent monitoring, and modular structural designs [19,20], yet systematic optimization remains lacking.

The findings revealed common issues: patients reported complicated operation, discomfort, monotonous training modes, and rigid actuation mechanisms; healthcare providers identified excessive weight, cumbersome interfaces, and lengthy recordkeeping processes; caregivers expressed concerns about financial burden and caregiving efficiency [7,9,17]. These insights highlight persistent gaps in functionality, user experience, and intelligent assistance that necessitate comprehensive optimization.

Globally, rehabilitation assistive technologies are evolving from purely mechanical systems toward intelligent interaction, modularity, and biomechanical adaptability. The integration of IoMT (Internet of Medical Things) is becoming increasingly important, and our device is designed with potential for seamless cooperation with IoMT ecosystems. This will allow the nursing bed to be integrated with broader healthcare networks, providing real-time data to caregivers and healthcare professionals and enabling personalized adaptive care for patients across different settings. Systems such as THERA-vital, Lokomat, and HAL-5 integrate multi-degree-of-freedom structural designs with muscle control [21,22,23,24,25,26,27,28]. In China, progress has been made in modular structures and human–machine interface optimization by institutions such as Yanshan University and Jilin University [29,30]. However, limitations remain in multi-scenario adaptability, precise lumbar support, and user emotion-responsive design.

In this context, we propose an intelligent optimization framework for the backrest-lifting nursing bed, aiming to deliver a low-cost, ergonomically optimized, lightweight, and interaction-friendly solution that improves applicability, safety, and user experience for people with movement disorders [14,15,16,18,21,22,23,24,25,26,27,28]. From an interdisciplinary perspective integrating rehabilitation medicine, mechanical engineering, and industrial design, a “behavior–emotion dual-channel data fusion” framework is developed, grounded in Norman’s emotional design theory to capture both explicit behavioral features and implicit emotional needs, and supported by evidence linking behavioral factors (e.g., logistics, packaging, after-sales service) with user emotions and satisfaction [2,3,4,5,28,31]. Methodologically, natural language processing and machine learning techniques (e.g., TF-IDF, LDA, Word2Vec + The K-Means (K-Means clustering) method) are employed to analyze user feedback, while the fuzzy Kano model is used for demand classification and prioritization, providing quantitative guidance for design optimization.

For engineering validation, the AnyBody Modeling System is used to simulate biomechanical interactions between the human body and the device during sitting–lying transitions. Analyses of muscle loads and joint moments inform the optimization of backrest angles, support structures, and mechanical conformity in the lumbar and dorsal regions. Furthermore, bionic curvature designs and adjustable backrest mechanisms are introduced to reduce shear forces and localized pressure; intelligent interaction modules, such as voice control and remote operation, simplify device operation; and gamified feedback mechanisms are implemented to enhance patient engagement and mitigate psychological stress.

Overall, this multidisciplinary, data-driven approach systematically enhances the functional performance and ergonomic adaptability of the backrest-lifting nursing bed, providing both a theoretical foundation and a practical design paradigm for the clinical application and personalized development of intelligent rehabilitation equipment.

## 2. Materials and Methods

The Behavior-Emotion Dual-Channel Framework integrates both functional and emotional dimensions of user needs to optimize product design. This framework consists of two key components: the behavioral layer, which addresses the user’s interaction with the product (e.g., purchase decisions, delivery, packaging, and after-sales service), and the emotional layer, which captures the emotional responses of users during their interaction with the product.

The behavioral layer of the framework focuses on the user’s behavior during the acquisition and post-purchase stages. It includes experiences related to logistics, packaging, and after-sales trust, which significantly shape user satisfaction. Although these factors do not directly pertain to the core functionality of the product, they influence the emotional response of the user, such as trust, attachment, and overall satisfaction. Previous studies indicate that logistics efficiency and packaging quality significantly affect the user’s first impression and emotional attachment to the product. Timely delivery and secure packaging not only contribute to a positive initial experience but also enhance trust and user satisfaction, thereby influencing their future behavior [32,33,34]. Additionally, the quality of after-sales service is a critical behavioral factor that directly impacts user trust. Positive after-sales experiences, such as responsive customer support and efficient return or repair services, foster a sense of security and emotional connection with the product and brand, improving long-term loyalty and satisfaction [35,36].

The emotional layer reflects how the product’s design meets the user’s emotional needs, such as comfort, ease of use, and functionality. By addressing both emotional and behavioral needs, this framework aims to enhance the overall user experience, ensuring that the product resonates on both a functional and emotional level. The interaction between these two layers captures the interplay where user behavior (e.g., purchase intent, repeat usage) and emotional responses (e.g., satisfaction, trust) continuously influence each other. Therefore, integrating both behavioral and emotional aspects provides a more holistic understanding of user needs, allowing for comprehensive product design optimization.

To validate this framework, we employ dual-channel data fusion, combining sentiment analysis from user reviews with biomechanical simulations (e.g., muscle load and joint stress analysis during posture transitions). This approach enables us to optimize the product design by considering both functional and emotional factors, ensuring that the nursing bed meets the user’s emotional and functional needs simultaneously.

### 2.1. Qualitative Study of the Backrest-Lifting Nursing Bed

#### 2.1.1. Sources and Collection Methods of User Review Data

The dataset used in this study was primarily obtained from major e-commerce platforms, with JD.com (www.jd.com) as the main source. JD.com is a comprehensive e-commerce platform providing one-stop shopping services, with a user base of several hundred million. Using the keywords “bed assistance device/machine” and “patient rehabilitation assistance bed”, products with top sales rankings were identified, and all available customer reviews for these products were selected as the research sample. Octopus Data Collector software was employed to perform web scraping, extracting four fields of information: username, review date, review text, and review URL. Review entries were retrieved and exported into an Excel file for subsequent analysis.

#### 2.1.2. Text Preprocessing and Chinese Word Segmentation Techniques

Raw text preprocessing is a critical step in the overall data preparation workflow, aiming to identify and rectify quality issues such as errors, missing values, and duplicate records, thereby enhancing data accuracy and consistency [37]. This process lays a solid foundation for subsequent data analysis and modeling, ensuring the scientific validity and reliability of the analytical results [38]. In this study, comments shorter than 10 characters, default positive feedback, duplicate entries, and blank texts were removed during preprocessing, resulting in a final dataset of 11,132 valid review records [38].

Following the data cleaning process, the Jieba segmentation tool, a Python-based Chinese word segmentation library, was employed to tokenize the review texts. Jieba combines statistical methods with rule-based matching to efficiently and accurately segment continuous Chinese text into semantically meaningful lexical units. In the subsequent text analysis, the tokenized results were transformed into analyzable lexical sets and applied to tasks such as word frequency statistics, sentiment analysis, and topic modeling, thereby providing essential foundational data for text mining.

#### 2.1.3. TF-IDF and Word Cloud Analysis

Key term extraction is a crucial step in text analysis, focusing on removing high-frequency words, prepositions, and punctuation marks that contribute little meaning while preserving the core semantic content of the text [39,40,41]. This process reduces noise and enhances the accuracy of tasks like sentiment analysis and topic modeling. In this study, a bilingual stop word list was used to filter out common, semantically minimal terms. The list, which consisted of 3076 words, was created by combining multiple authoritative sources.

**Figure 1:**
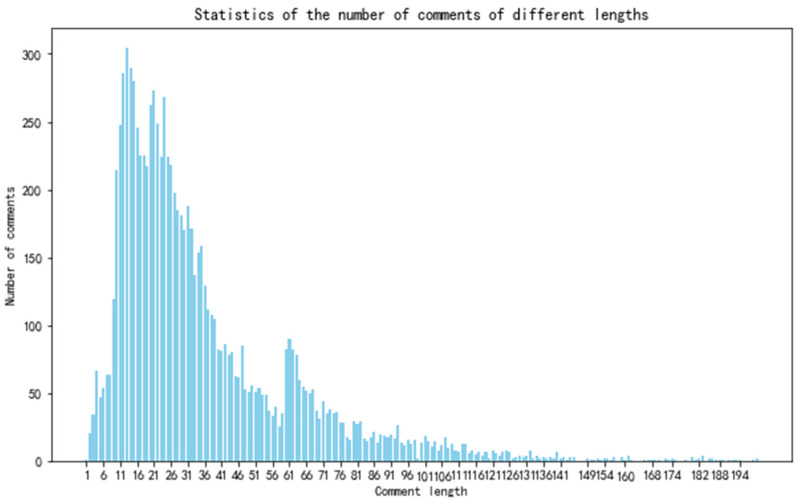
Statistical distribution of the number of user comments with a length of less than 200 characters.

**Figure 2:**
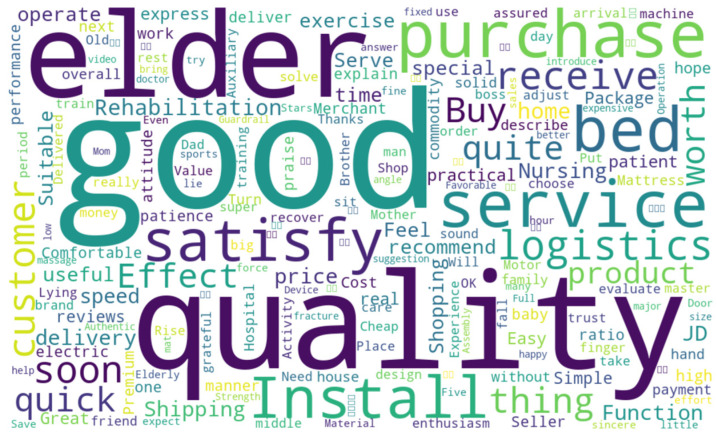
Word cloud of comment data.

A review length distribution analysis revealed that 99.5% of reviews were under 200 characters (Figure 1) displaying a typical long-tail pattern. Short reviews were more common, while longer reviews, providing detailed product feedback, were less frequent. High-frequency terms like “quality,” “good,” “elderly,” “product,” and “function” dominated, reflecting user concerns about product quality and functionality. Positive sentiments were indicated by terms such as “satisfactory,” “easy to use,” and “worthwhile,” while functional terms like “installation,” “operation,” and “speed” showed a focus on ease of use and caregiving. These insights guide product optimization, such as improving installation simplicity and caregiving features. The word cloud of the comment data is shown in Figure 2.

After Chinese word segmentation (Jieba), lower-casing, punctuation stripping, and stop-word removal, the study computed term salience using the standard TF-IDF weighting scheme rather than reproducing formulas. Concretely, we used raw within-document term counts with sublinear scaling (applied only to non-zero counts), an inverse-document-frequency (IDF) computed with a natural logarithm and +1 document-frequency smoothing to avoid division-by-zero, and L2 normalization at the document level to mitigate length effects.

For corpus-level keyword ranking, we aggregated per-document TF-IDF weights either by the maximum or the mean value across all documents; the top-ranked tokens are reported in Table 1, which subsequently feed topic clustering and requirement mapping in Section 2.2. This configuration follows widely adopted practice in information retrieval and text mining and ensures reproducibility without burdening the reader with standard equations [42].

### 2.2. Quantitative Research on the Backrest-Type Lifting Nursing Bed

#### 2.2.1. Analysis and Classification Using the Latent Dirichlet Allocation (LDA) Topic Model

Latent Dirichlet Allocation (LDA) is a widely used topic modeling algorithm for uncovering latent thematic structures in large-scale text datasets. LDA assumes that each document is a mixture of several topics, with each topic represented by a specific probability distribution over words. By inferring the topic distribution of documents and the word distribution of topics, LDA is capable of identifying hidden semantic patterns within the text. The method is based on a generative probabilistic model, in which the topic distribution of a document follows a Dirichlet distribution, and word generation is determined by topics. Inference is typically performed using Gibbs sampling or Variational Inference.

In model evaluation, perplexity is a commonly used metric for assessing the quality of an LDA model, reflecting its predictive performance on unseen data. A lower perplexity value indicates a better fit to the data, as it is computed based on the likelihood of generating the test documents and represents the model’s average uncertainty in text generation [43,44]. However, excessively low perplexity may lead to overfitting or poorer human-perceived topic quality, so model selection should balance perplexity with qualitative/semantic coherence [45,46]. By examining the relationship between the number of topics and perplexity, this study selected Topics = 8, as it yielded a low perplexity without evident overfitting.

Using this configuration, the LDA model was applied to cluster online review data into topics. Each topic was then semantically categorized according to its representative keywords, resulting in eight distinct thematic categories (Table 2).

#### 2.2.2. Clustering Analysis of Text Data

Word2Vec, proposed by Google, is a word-embedding method that maps words into dense low-dimensional vectors, thereby capturing semantic similarity and contextual relationships [47,48]. Two commonly used models are Continuous Bag of Words (CBOW), which predicts the target word from its context, and Skip-gram, which predicts the context from the target word [49]. Word2Vec is recognized for its strong semantic representation capability and computational efficiency. K-Means, on the other hand, is a classical unsupervised clustering algorithm that partitions data into K clusters by minimizing within-cluster variance through iterative optimization. It is simple to implement and computationally efficient, though sensitive to initial cluster centers. When combined, Word2Vec provides high-quality semantic representations, while K-Means clusters these embeddings to reveal latent text patterns. In applications such as product review analysis, this approach can map user comments into vectors and group them into distinct themes (e.g., product quality, logistics, or Maintenance turnaround and spare-part availability). Similarly, in customer feedback analysis, clusters can be aligned with different sentiment or requirement categories, offering valuable insights for user-centered product optimization.

In this study, we applied the Word2Vec + K-Means approach to cluster product review data from the JD.com platform. Word vectors were first generated using Word2Vec, followed by K-Means clustering, resulting in five clusters. For each cluster, representative keywords and thematic categories were extracted (Table 3).

#### 2.2.3. Mapping of User Review Topics to Design Requirements

The mapping of themes to user needs is one of the core analytical aspects of this study for further development. This step aims to accurately associate keywords and topics derived from user reviews with specific user requirements, thereby transforming surface-level review content into deeper-level demand insights. Specifically, the frequency statistics of online reviews and the characteristic terms of each topic were integrated to construct a mapping between the features of bed-assistance and rehabilitation devices and corresponding user needs. The resulting mapping provides explicit guidance for product optimization and ensures that the design better aligns with actual user requirements. The mapping results are presented in Table 4.

### 2.3. Classification and Prioritization of User Requirement Attributes

#### 2.3.1. Principle of the Fuzzy Kano Model and Questionnaire Design

The fuzzy Kano model is based on fuzzy set theory proposed by Professor Zadeh at the University of California, which quantifies fuzzy attitudes using interval fuzzy numbers [0, 1]. This approach effectively accommodates users’ inherent cognitive fuzziness in decision-making and is particularly suitable for situations where respondents exhibit uncertainty in attitude selection [50]. Compared with the traditional Kano model, the fuzzy Kano model offers a more refined scoring mechanism, capable of capturing subtle variations in user psychology and accounting for inter-group differences, thereby enhancing the accuracy and reliability of the analysis.

In this study, the fuzzy Kano model was applied to quantitatively analyze user requirements, with a questionnaire designed to incorporate both positive and negative perspectives. Respondents were allowed to assign multiple attitude values to both positive and negative options, with the sum of the assigned values constrained to 1, thereby reflecting their relative preferences and trade-offs. The questionnaire format for the fuzzy Kano model is presented in Table 5.

#### 2.3.2. Fuzzy Kano Classification Evaluation and Result Analysis

Taking the safety requirement “adjustable resistance” as an example, the calculation procedure for user requirement membership degrees in the fuzzy Kano model is illustrated as follows.

(1) Data Collection

A total of 285 questionnaires were distributed. After excluding 16 invalid responses, 269 valid samples were retained. Table 6 presents a subset of the collected data as an example.

(2) Data Calculation

Based on the responses provided by the user, the “presence” matrix and “absence” matrix were constructed, from which the fuzzy interaction matrix W was derived:

(1) $$W=X^{T}Y\left[\begin{array}{ccc} \begin{array}{c} 0\\ 0 \end{array} & \begin{array}{c} \begin{array}{cc} 0 & 0 \end{array}\\ \begin{array}{cc} 0 & 0 \end{array} \end{array} & \begin{array}{cc} 0.12 & 0.48\\ 0.08 & 0.32 \end{array}\\ \begin{array}{c} 0\\ 0 \end{array} & \begin{array}{c} \begin{array}{cc} 0 & 0 \end{array}\\ \begin{array}{cc} 0 & 0 \end{array} \end{array} & \begin{array}{cc} 0 & 0\\ 0 & 0 \end{array}\\ 0 & \begin{array}{cc} 0 & 0 \end{array} & \begin{array}{cc} 0 & 0 \end{array} \end{array}\right]$$

By mapping the fuzzy interaction matrix to the fuzzy Kano model demand classification evaluation table, the membership degree vector Z for the requirement element “adjustable resistance” can be obtained:

(2) $$Z=\left(\frac{M}{0.32},\frac{O}{0.48},\frac{I}{0.08},\frac{A}{0.12},\frac{R}{0}\right)$$

(3) Attribute Classification

From the above results, the membership degree vector Z indicates that the “adjustable resistance” requirement is associated with four categories: M, O, I, and A. To determine the final classification, a confidence level was introduced as a filtering criterion. Based on prior research, a threshold of λ = 0.4 was selected as the appropriate confidence level for category determination, which has been widely used in previous fuzzy Kano studies and shown to balance classification stability and interpretability [50]. Comparing the membership degrees with this threshold, the “adjustable resistance” requirement was ultimately classified as a One-dimensional (O) requirement. The Fuzzy Kano Model Demand Attribute Classification Evaluation Table is shown in Table 7.

(4) Attribute Determination

For the given requirement element, Steps (1) to (3) were repeated for all survey respondents. The classification tendency of each respondent toward the requirement element was recorded, and the category with the highest frequency of occurrence across all respondents was assigned as the final classification for that requirement element.

Following the categorization method of the fuzzy Kano model, the classification results for all user requirement elements were organized and grouped into six dimensions: safety, ergonomics, interaction friendliness, product appearance design, adaptability, and after-sales and maintenance (Table 8).

In the subsequent analysis, requirement elements that had no significant impact on product design, such as Reverse-type requirements (R) and Questionable requirements (Q), were excluded. The aggregated requirement attribute classification results are presented in Table 9.

The results indicate that, based on the fuzzy Kano model, the primary categories of product and service requirements are One-dimensional requirements (O), Must-be requirements (M), Attractive requirements (A), and Indifferent requirements (I).

#### 2.3.3. Better–Worse Satisfaction Coefficient Evaluation

After determining the attribute classification of each service requirement, the Better–Worse coefficient can be calculated to establish the priority order for service provision. The Better–Worse coefficient evaluates the extent to which the provision or absence of a service influences user satisfaction and dissatisfaction.

The Better coefficient is typically positive, indicating that providing the service will increase user satisfaction, with larger values representing faster satisfaction growth. The Worse coefficient is typically negative, indicating that the absence of the service will decrease user satisfaction, and a larger absolute value corresponds to a faster rate of dissatisfaction growth. The formulas for calculating the Better–Worse coefficients are as follows:

(3) $$Better=\frac{A+O}{A+O+M+I}$$

(4) $$Worse=-1\times \frac{O+M}{A+O+M+I}$$

The satisfaction coefficients corresponding to the 35 service attributes identified in this survey were calculated, and the results are presented in Table 10.

Based on the classification results of the fuzzy Kano model, the Better–Worse coefficients for each service item were calculated, and the demand hierarchy was analyzed according to the service requirement categories. Following the priority ranking rules of the fuzzy Kano model, service requirements were prioritized as follows: Must-be (M) > One-dimensional (O) > Attractive (A) > Indifferent (I). Within the same requirement category, services with higher Better coefficients were given higher priority.

#### 2.3.4. Extraction of Key Design Elements for Lifting and Lowering Aids

**Figure 3:**
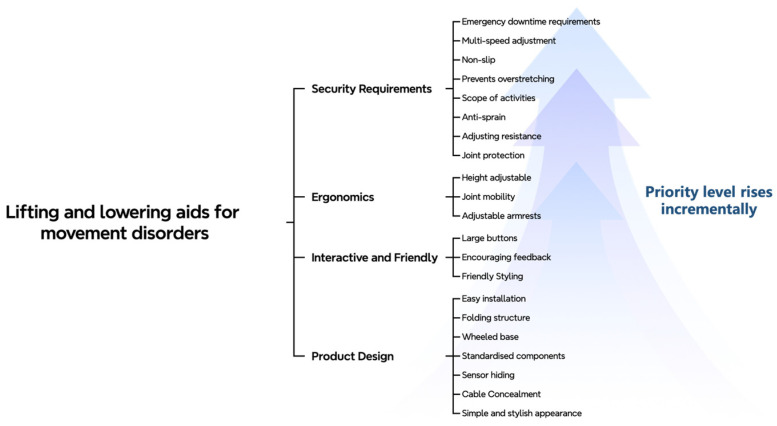
Design elements of a backrest-lifting nursing bed.

User requirements were extracted using online text mining techniques, and the identified requirements were subsequently classified and prioritized through the fuzzy Kano model. By integrating the outcomes of online text mining, user requirement analysis, and product feature prioritization, innovative design solutions were developed for multiple aspects of the bed-assistance device. Three major categories of core requirements were identified: overall appearance requirements, functional requirements, and human–machine interface requirements. Based on these, an overall design solution for the bed-assistance device was proposed, and the key design elements were distilled. The design elements of the bed-assistance device are illustrated in Figure 3. The optimization items in Section 2.4 directly operationalize the retained requirement attributes summarized in Table 9 (Retain), with IDX codes cited per item to ensure traceability from need to design implementation.

### 2.4. Backrest Lift Nursing Bed Product Design

#### Product Design Optimization Points for Backrest Lifting Nursing Beds

Based on user requirement mapping and priority analysis, this study optimized the bed-assistance device in four aspects: safety, ergonomics, interaction experience, and product appearance design. For safety, a low-center-of-gravity frame, anti-slip pads, anti-loosening locking structures, and an inclination angle sensor were incorporated to enhance stability and operational safety. Ergonomic improvements included multi-angle adjustable backrest and leg supports, as well as optimized support distribution to improve ergonomic fit and reduce peak back-muscle loading during posture transitions. The design of the nursing bed was based on user needs and biomechanical simulations. A comparative analysis was performed against relevant the International Organization for Standardization (IEC) standards, such as IEC 60601-2-52: Medical beds—Part 2–52 for safety and performance of medical beds, as well as several existing commercial nursing beds. These comparisons focused on key factors such as functionality, safety, comfort, and adjustability, to highlight the advantages of the proposed design in meeting user needs. In appearance design, lightweight high-strength materials and breathable eco-friendly cushions were used, combined with a modular structure to improve portability and maintenance.

**Figure 4:**
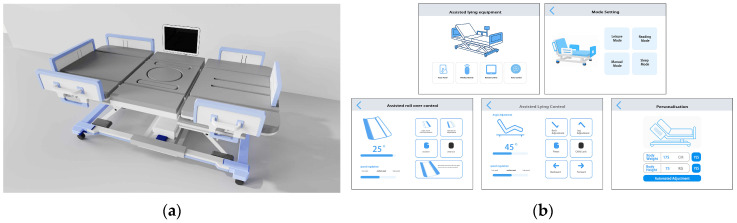
Product rendering and smart interaction interface of the back-support lifting nursing bed (**a**) Product rendering: Shows the overall structure and functional layout of the bed, incorporating optimized design elements such as a low-center-of-gravity frame, anti-slip base, multi-angle adjustable backrest and leg supports, breathable eco-friendly cushions, and modular construction; (**b**) Smart interaction interface: Displays the mobile app interface for remote control and mode selection, supporting one-touch sit/lie transitions, fine angle adjustments, status monitoring, and personalized parameter settings to enhance user operation experience and improve the device’s level of intelligence.

Building on the optimization strategies and design elements described above, we developed the final design scheme for the back-support lifting nursing bed; its overall structure and functional layout are shown in Figure 4. This figure clearly demonstrates the integration of the optimized design measures within the product, encompassing safety-oriented structural enhancements, ergonomic adjustments, interactive feature configurations, and aesthetic improvements. These design implementations collectively address core user requirements while ensuring comfort, safety, and a high level of intelligence.

Following the development of the product renderings for the back-support lifting nursing bed (see Figure 4), a systematic summary of the optimization strategies is presented in Table 11. This table consolidates the design improvements across structure, safety, interaction, materials, and sustainability, detailing the technical implementations and anticipated benefits. By providing this comprehensive overview, the table not only enables readers to quickly grasp the design highlights but also serves as a clear technical reference for subsequent AnyBody simulation analysis and structural performance validation.

**Table 11:** Summary of Comprehensive Optimization Strategies for the Back-Support Lifting Nursing Bed.

Optimization Aspect	Technical Implementation	Expected Outcome
Modular Structure Design	Separation of four major modules—main frame, support system, control system, and interaction system—connected via detachable joints	Facilitates transportation, maintenance, and upgrades, extending product life cycle
Low-Center-of-Gravity Stable Frame	Forward-tilted structure (5°) + bed base widened by 10 cm	Reduces tipping risk and enhances stability on various floor types
Anti-Slip and Anti-Tip Structures	Self-locking casters (automatic/manual switch) and intelligent anti-tip alarm system (>10° tilt alarm + automatic adjustment)	Improves operational safety, preventing sliding and tipping
Assisted Turning Mechanism	Electric tilting panels, adjustable lumbar support, and timed automatic turning	Reduces pressure sore risk and enhances comfort for long-term bedridden users
Multi-Modal Interaction	AI voice assistant, physical control buttons, and smartphone APP control	Enhances operational convenience, accommodating diverse user preferences
Personalized Posture Adjustment	Electrically adjustable backrest (0–90°) and leg support (0–45°) with automatic optimal angle calculation	Improves ergonomic fit and reduces peak erector-spinae loading during mid-to-low recline (60°→30°→0°) as indicated by Section 3.3, enhancing comfort during sit-to-supine transitions.
Lightweight High-Strength Structural Materials	Aluminum alloy (6061-T6), carbon fiber composites, and stainless-steel key components	Reduces weight (approx. 25–30 kg) while maintaining durability and load capacity
Comfort-Oriented Contact Materials	Memory foam, high-elastic PU foam, and medical-grade breathable fabric	Minimizes localized pressure and heat build-up, improving long-term comfort
High-Precision Manufacturing Processes	Laser cutting + CNC machining, robotic welding, in-mold injection, and eco-friendly powder coating	Improves structural accuracy and corrosion resistance, extending service life
Eco-Friendly and Sustainable Design	Recyclable materials, low-VOC coatings, and modular replaceable components	Designed to meet RoHS requirement standards, reduces maintenance costs, and enhances market competitiveness

Optimization Aspect represents the design dimension of improvement; Technical Implementation refers to the control measures; Expected Outcome indicates the achieved functionality. Note: Each Expected Outcome is grounded in retained fuzzy-Kano attributes (Table 9) and/or Section 3 biomechanical findings; see the traceability table (Table 12) for IDX-level mapping.

**Table 12:** Traceability from retained requirements (Table 9) to Section 2.4 optimizations.

Optimization Aspect	Mapped Retained Attributes (IDX → Feature)	Evidence Pointer
Low-center-of-gravity frame, anti-slip/anti-tip	A6 (Antiskid, M), A8 (Emergency shutdown, M), D2 (Wheeled base, M)	Design rationale in 2.4; safety set in Table 9; cf. stability emphasis in Section 3 scenarios.
Personalized posture adjustment (0–90° backrest; 0–45° leg support)	B1 (Height adjustable, O), B3 (Full joint range of motion, O), A7 (Joint protection, A)	Back-muscle load patterns across 90° → 0°; ES load decreases <60°.
Assisted turning mechanism and adjustable lumbar support	A3 (Range of activity, O), A4 (Prevent overstretching, O), A5 (Prevent sprains, O)	Pressure distribution and support needs in S1–S4 scenarios (Section 3).
Multi-modal interaction (buttons/APP/voice)	C1 (Large-sized buttons, M), C3 (Encouraging feedback, O), E2 (High contrast, O), E3 (Language, O), E4 (Voice, M)	Reduces pressure sore risk and enhances comfort for long-term bedridden users
Multi-Modal Interaction	AI voice assistant, physical control buttons, and smartphone APP control	Usability/operation priorities retained in Table 9.
Modular structure and maintainability	D1 (Folded structure, M), D3 (Sensor hidden, O), D4 (Cable concealment, O), D8 (Normalizer, O), F5 (Simple installation, M), F4 (Maintenance turnaround and spares, A)	Maintainability priorities retained in Table 9; see Table 11 for implementations.

Note: The term “muscle fatigue” is replaced by “peak muscle loading” to reflect the measurable biomechanical quantity reported in Section 3.

On this basis, the AnyBody simulation platform will subsequently be employed to validate the biomechanical performance of the product under representative usage scenarios, with a focus on analyzing muscle load distribution and joint force characteristics. The results will be used to further refine the structural design and parameter configuration, ensuring that the final solution aligns more closely with ergonomic principles and the practical needs of target users.

## 3. Experiment and Result Analysis

### 3.1. Man-Machine Integration Modeling

In the design and development of rehabilitation assistive devices, accurately evaluating the biomechanical effects of a product on the human body is crucial for ensuring safety and enhancing comfort. In this study, AMS (the AnyBody Modeling System) was employed to establish a human–machine integrated model for the designed back-support lifting nursing bed, providing a solid foundation for subsequent kinematic and dynamic analyses.

First, the three-dimensional structural model of the device, comprising the seat base, backrest, armrests, leg supports, and footrests, was developed in Rhino software. The model was exported in ASCII-format. These components were then imported into the Environment Model module of AMS, where their initial positions were defined to align with the spatial configuration of the human model.

While the human model used in this study was based on the GENERICBODYMODEL template, which offers a standard representation of the skeletal and muscular system, we acknowledge that this model does not account for key anthropometric variables such as Body Mass Index (BMI) and sex. These factors could influence muscle mass distribution and joint angles, potentially affecting the biomechanical outcomes. However, for the purposes of this study, the use of the GENERICBODYMODEL template provided a reasonable approximation for the general user population. Future work should incorporate personalized models that adjust for BMI and sex to enhance simulation accuracy and better represent diverse user profiles. To adapt the model to the initial sitting posture required for the back-support lifting nursing bed, the Mannequin. Posture parameters were refined, including adjustments to pelvis height (PelvisPosY), pelvis rotation angle (PelvisRotX), and limb positioning, ensuring precise alignment between the human model’s posture and the device’s structure.

Coupling between the device and the human body was achieved through spatial reference nodes (AnyRefNode) and multiple joint constraints (AnyStdJoint, AnyRevoluteJoint, AnyPrismaticJoint). Reference points were established on key parts of the seat, backrest, armrests, leg supports, and footrests and aligned with the corresponding skeletal contact points of the human model to ensure consistency between force application points and actual contact areas. The constraints restricted relative motion between the human body and the device, including pelvis–seat fixation, back–backrest contact, foot–footrest support, hand–armrest support, and backrest rotation adjustment.

To replicate a natural sit-to-stand transition, motion drivers were defined in the JointsAndDrivers.any file, covering backrest elevation, knee flexion, pelvis translation, and adjustments to foot and hand positions. These drivers ensured that the device’s motion trajectory was consistent with natural human movement patterns.

**Figure 5:**
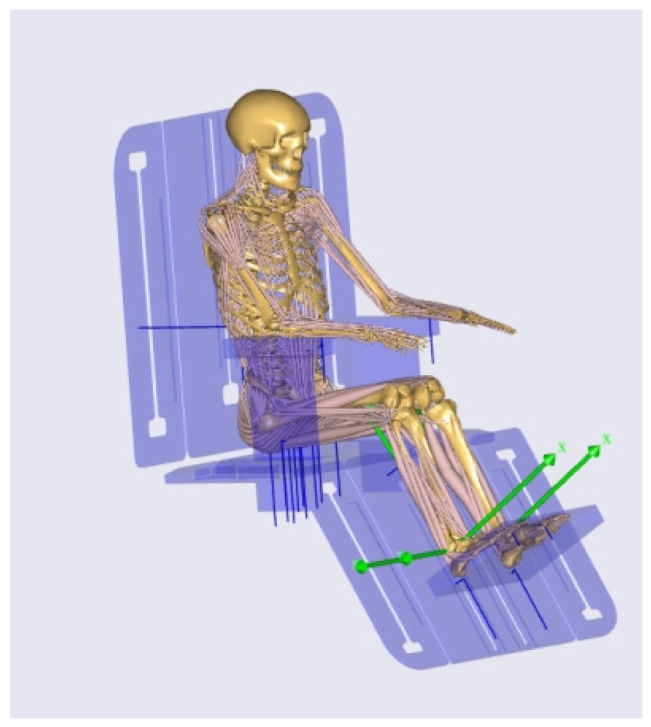
Human–machine integrated model of the back-support lifting nursing bed.

Through this modeling process, the human–machine integrated model shown in Figure 5 was established. This model accurately reproduces postural changes and contact relationships during product use, providing a reliable basis for subsequent kinematic, dynamic, and muscle load analyses.

### 3.2. Simulation Scenarios for the Use of Backrest Lifting Nursing Beds

To comprehensively evaluate the biomechanical impact of the sit-to-lie assistive device on users, this study established a complete human–machine simulation environment in the AnyBody Modeling System (AMS) to replicate the key motion process from a fully seated to a supine posture. Stage-by-stage simulations were conducted to analyze muscle load distribution, joint force characteristics, and potential fatigue risks under different postures, with the aim of optimizing the device’s support structure and assistive functions. The simulation process was divided into four representative scenarios, each corresponding to a specific user posture and associated biomechanical measurements.

#### 3.2.1. S1: Fully Seated (90°)

In the initial stage, the user maintains an upright seated position, with the back in full contact with the backrest and the lower limbs naturally flexed. This stage primarily assesses pressure distribution on the seat cushion and buttocks, as well as lumbar support effectiveness, ensuring prolonged sitting does not induce discomfort or fatigue. Given that prolonged sitting may cause sustained tension in the erector spinae and quadratus lumborum muscles, lumbar support adequacy was evaluated, along with the stabilizing role of armrests during posture adjustments. If localized pressure concentrations were detected in the buttocks or lumbar region, modifications such as optimized seat cushion padding or adjustable lumbar support were recommended.

#### 3.2.2. S2: Reclined to 60°

When the backrest reclines to 60°, the user transitions into a semi-reclined posture. This stage focuses on the load variations in the erector spinae and multifidus muscles, with special attention to peak spinal shear forces at this angle. The device must provide sufficient structural support to alleviate muscular load and ensure proper load distribution in the hip and knee joints. In cases of excessive muscle loading or inadequate lumbar support, improvements to the backrest cushioning system were suggested to reduce impact forces.

#### 3.2.3. S3: Reclined to 30°

With the backrest further lowered to 30°, the user approaches a near-supine position, resulting in significant changes in the load distribution across the shoulders, back, and lumbar region. This stage emphasizes spinal pressure distribution and head–neck support adequacy to prevent discomfort due to insufficient support. As the 15–45° range is a critical phase of heightened back muscle loading, effective support at the neck, lumbar, and back contact points is essential. If concentrated loads were identified in these areas, backrest curvature optimization or the addition of an adjustable headrest was advised.

#### 3.2.4. S4: Fully Supine (0°)

In the final stage, the user adopts a fully supine posture, maximizing the body’s contact area with the bed surface. This stage examines pressure distribution over the back, buttocks, and shoulders to prevent discomfort or pressure ulcer risks caused by localized load concentrations. If high pressure was detected in the buttocks or shoulder regions, enhancements to mattress cushioning or the integration of lumbar support structures were proposed.

The four-scenario simulation framework provides a comprehensive biomechanical evaluation of the device across different usage stages, offering quantitative evidence and theoretical guidance for subsequent structural optimization and parameter adjustments.

### 3.3. Analysis of Key Biomechanical Parameters

#### 3.3.1. Analysis of Muscle Loading During Sitting–Lying Position Transitions

**Figure 6:**
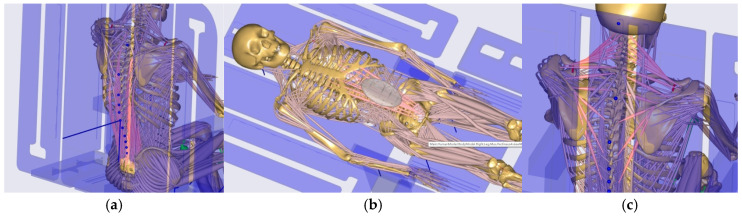
Anatomical visualization of the primary muscle groups. (**a**) Erector Spinae; (**b**) Rectus Abdominis; (**c**) Trapezius. The corresponding muscles are highlighted in pink to clearly illustrate their spatial distribution and morphological characteristics within the human body.

To evaluate the support performance and muscle loading characteristics of the back-support lifting nursing bed during posture transitions, the AnyBody simulation platform was used to analyze the complete process from full sitting (90°) to semi-reclined positions (60°, 30°) and finally to supine (0°). The analysis focused on the erector spinae, rectus abdominis, and trapezius muscles. For clarity, the anatomical locations of these three muscle groups were extracted and visualized in the simulation (Figure 6), providing a clear spatial representation and morphological reference for subsequent force curve analysis.

(1) Erector Spinae

In the 90° → 60° range, the erector spinae exhibited substantial loading, particularly in the T11-L1 and T9-T10 segments, with a peak force of approximately 6 N, indicating its role in maintaining spinal stability and supporting upper body weight. During the 60° → 30° phase, loading decreased significantly in regions such as T11-Sacrum and L2–L3, reflecting effective backrest support; however, L1–L2 retained a moderate load, suggesting partial involvement of the back muscles. In the 30° → 0° phase, most erector spinae segments approached 0 N, indicating full weight support by the device, except for minor residual loads in L4–L5 and L5–S1, potentially due to localized support deficiencies.

(2) Rectus Abdominis

The rectus abdominis demonstrated low loading from 90° to 30°, but showed a pronounced increase during the 30° → 0° transition. This indicates its role in controlling deceleration and preventing abrupt posture changes during the final reclining phase, especially when the backrest lowers rapidly.

(3) Trapezius

The trapezius exhibited regional differences in loading patterns:

Clavicular region: At 90°, loading was minimal (<4 × 10^−11^ N) but increased markedly during the 60° → 30° phase (peak 1.4 × 10^−10^ N), reflecting its involvement in shoulder stabilization. From 30° → 0°, loading decreased rapidly toward 0 N, with only minimal residual forces (~2 × 10^−11^ N) in certain cases.

Scapular region: Initial loading was low (2 × 10^−12^ N to 3 × 10^−12^ N), increased progressively in the 60° → 30° range, peaking at 5 × 10^−12^ N, indicating its critical role in maintaining shoulder stability in mid-range inclinations. In the 30° → 0° phase, loading dropped sharply, though slight increases were observed after 0.75 s, possibly due to uneven shoulder support distribution.

**Figure 7:**
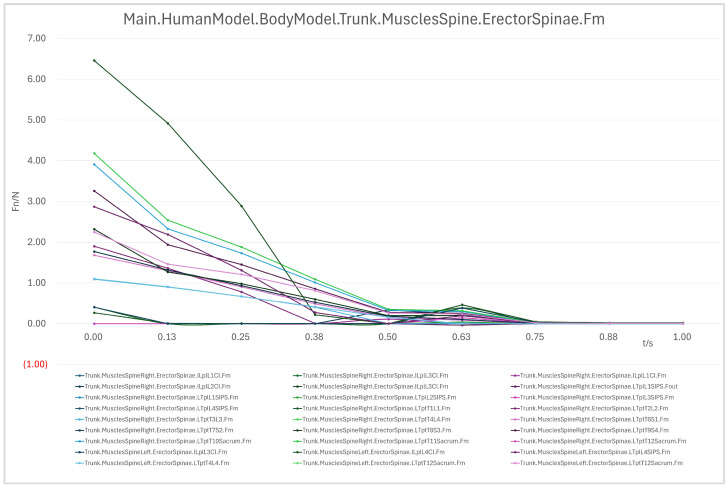
Erector Spinae Force Analysis.

**Figure 8:**
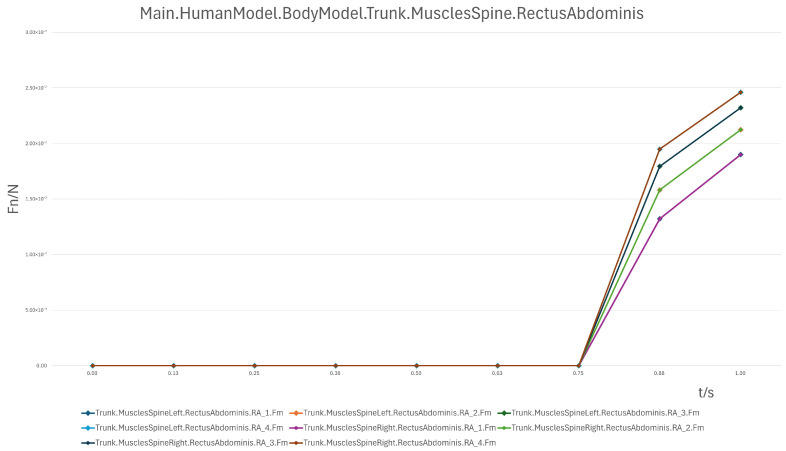
Rectus Abdominis Force Analysis.

**Figure 9:**
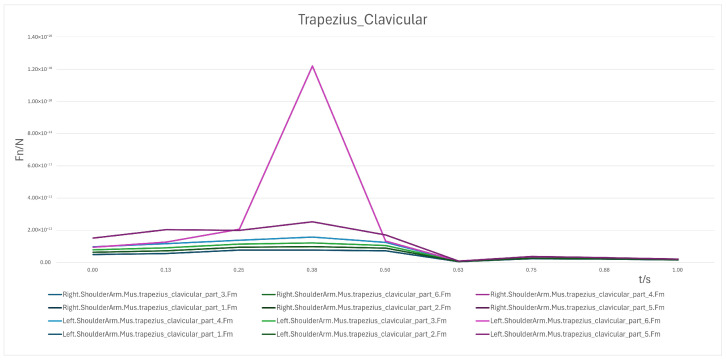
Clavicular Trapezius Force Analysis.

**Figure 10:**
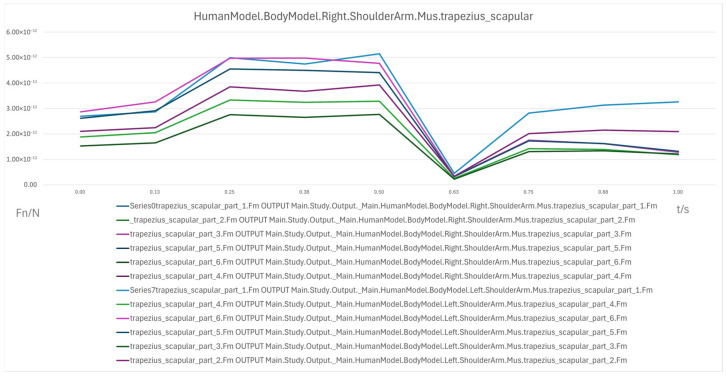
Scapular Trapezius Force Analysis.

In summary, the erector spinae primarily support the back during the initial sitting phase, the rectus abdominis experiences a transient load increase in the final reclining phase, and both the clavicular and scapular portions of the trapezius muscles contribute to shoulder stability control during the mid-transition phase. To visually illustrate these load variation characteristics, Erector spinae force analysis: illustrates force variations across spinal segments during the 90° → 0° transition, reflecting changes in back support and spinal stability (Figure 7); Rectus abdominis force analysis: shows transient load increase in the core abdominal muscles during the final reclining phase (30° → 0°) (Figure 8); Clavicular trapezius force analysis: highlights peak force variations in shoulder-stabilizing muscles during the mid-transition phase (60° → 30°) (Figure 9); Scapular trapezius force analysis: depicts changes in shoulder stability and localized support differences during mid-transition and final supine positioning (Figure 10).

#### 3.3.2. Analysis of the Forces on the Transition Joints in the Sitting and Lying Positions

During the sit-to-stand and recline-to-supine transitions, the hip and knee joints play a critical role in posture control and load distribution, exhibiting distinct force characteristics at different stages. To systematically evaluate these variations, three user models with different statures (160 cm, 175 cm, 185 cm) were analyzed, with joint reaction forces recorded at four posture stages (90° → 60° → 30° → 0°) and directional components assessed.

##### Hip Joint Load Analysis

**Figure 11:**
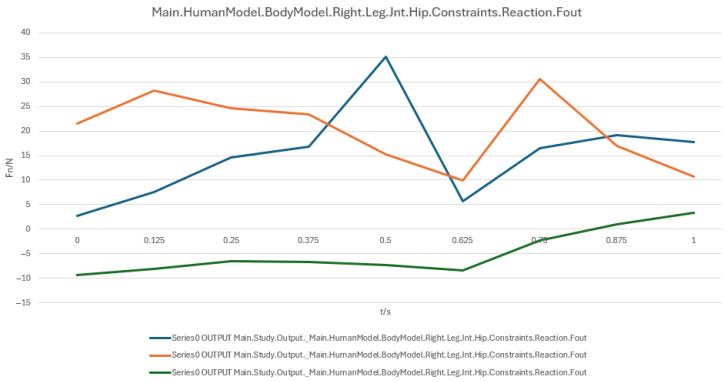
Right hip joint reaction force.

**Figure 12:**
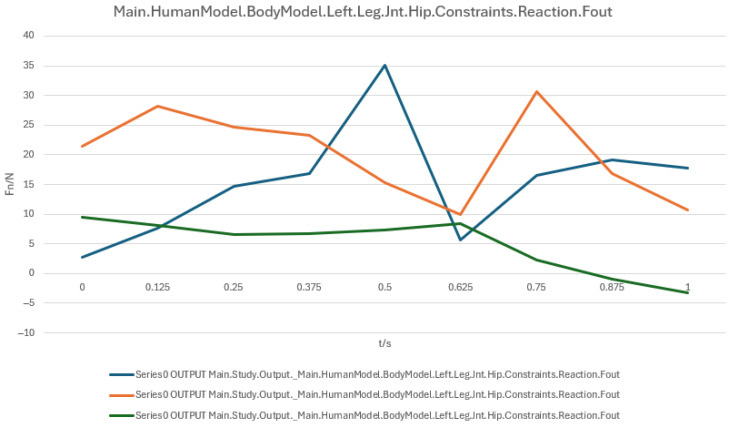
Left hip joint reaction force.

The reaction forces of the right and left hip joints are shown in Figure 11 and Figure 12, respectively. Overall, the hip joint loads demonstrated a fluctuating pattern with substantial differences among directional components. At approximately 0.5 s, the blue curve (primary load component) peaked at ~30 N, indicating a critical moment in posture transition when the hip joint compensates for insufficient device support. The orange curve showed relatively stable variations, suggesting even load distribution in certain directions, while the green curve exhibited low magnitudes, indicating a minor contribution to total load. Key time intervals were as follows:

0.125 s~0.5 s: Gradual load increase as the hip joint supports body weight and adjusts posture;

0.5 s (Peak): Load spike possibly due to abrupt changes in support;

0.625 s: Significant load drop, suggesting activation of cushioning mechanisms;

0.875 s~1.0 s: Load stabilization as the user reaches the fully supine position.

##### Knee Joint Load Analysis

**Figure 13:**
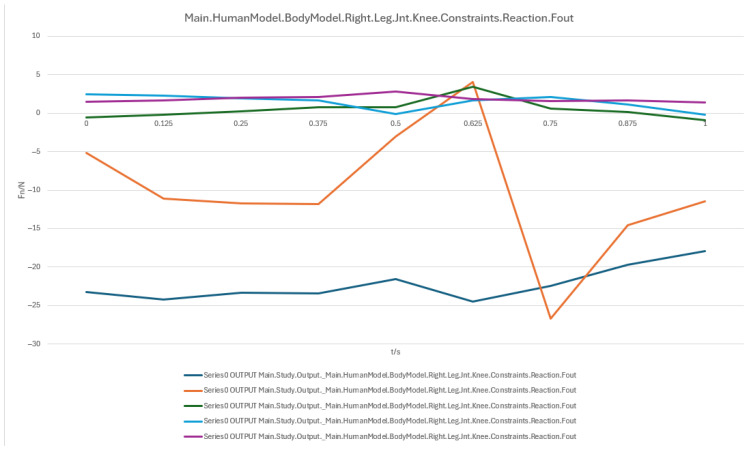
Right knee joint reaction force.

**Figure 14:**
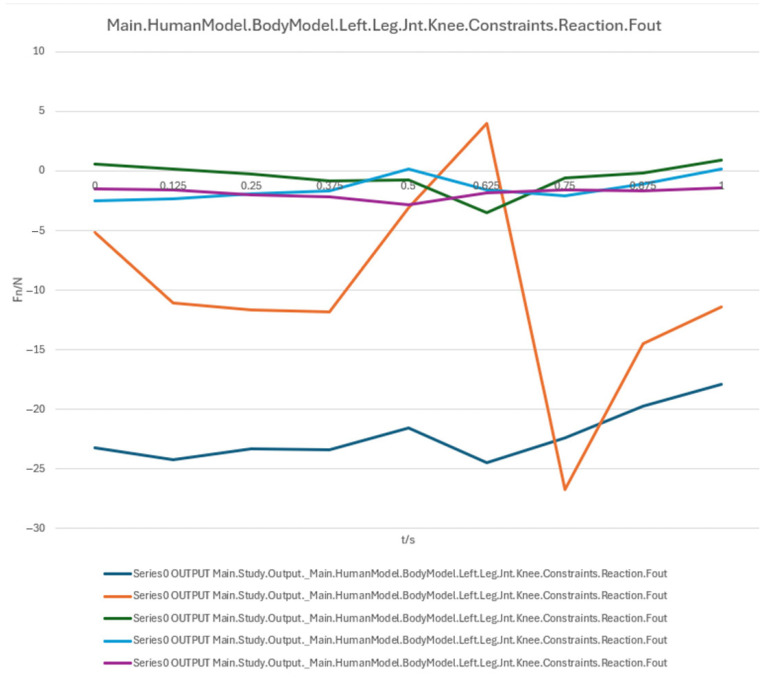
Left knee joint reaction force.

The reaction forces of the right and left knee joints are shown in Figure 13 and Figure 14, respectively. During the initial phase (0 s~0.5 s), knee joint loads increased steadily, with the blue curve peaking at ~35 N at 0.5 s, followed by a sharp drop to near zero before 0.625 s, likely due to a shift in the center of gravity or a change in device support mode. The orange curve remained stable, with minor fluctuations around 0.75 s potentially caused by trunk adjustments or secondary device support, while the green curve remained minimal throughout and approached zero, indicating high stability in the horizontal force component. Key time intervals were as follows:

0.5 s (Peak): Critical stage of posture change, where insufficient support may lead to sudden load spikes;

0.625 s (Drop): Cushioning or weight-shift phase, requiring smooth control of support angle adjustments;

0.75 s (Secondary fluctuation): Possibly related to lower limb repositioning or secondary device support response.

In summary, both hip and knee joints exhibit a peak–cushion–stabilization load pattern, highlighting the need for optimized support and cushioning systems during the mid-transition phase to reduce instantaneous joint loads and enhance comfort and safety. To facilitate direct comparison and trend evaluation, the variation in hip and knee joint reaction forces during the sit-to-supine transition is shown: Right hip joint reaction force (Figure 11); Left hip joint reaction force (Figure 12); Right knee joint reaction force (Figure 13); Left knee joint reaction force (Figure 14).

## 4. Discussion

### 4.1. Discussion of Innovative Features

Rehabilitation technologies have progressed from primarily mechanical assistance toward intelligent interaction, modularity, and biomechanical adaptability. Representative systems such as THERA-vital, Lokomat, and HAL-5 integrate multi-DOF structures with muscular control and have demonstrated measurable rehabilitation benefits across gait and limb support tasks [21,22,23,24,25,26,27,28]. Building on this trajectory, recent community efforts in China have advanced modular structures and human–machine interface optimization for assistive devices. Our work aligns with this evolution but differs in three ways: (i) we fuse a behavior–emotion dual-channel pipeline grounded in user-generated reviews to expose latent experiential demands; (ii) we couple these demands to AnyBody-based biomechanical simulations that quantify muscle and joint responses across posture transitions; and (iii) we architect the device with IoMT-ready connectivity to enable data sharing and adaptive care pathways across settings, as outlined in the introduction. Together, these elements provide human-centered, and data-driven optimization route that complements and extends prior robotic and medical-bed solutions and is consistent with the manuscript’s earlier positioning of IoMT integration and state-of-the-art exemplars.

Furthermore, beyond functional benchmarking, the present design was compared against IEC medical-bed safety/performance requirements and existing commercial products, revealing advantages in adjustability, comfort, and user-centric interaction. We surface this comparison here to situate the contribution among regulatory and market baselines rather than only within engineering results.

### 4.2. Key Biomechanical Findings and Design Implications (Drop-In Replacement)

This study employed the AnyBody Modeling System to conduct a comprehensive biomechanical analysis of a back-support lifting nursing bed during the transition from sitting to lying. We examined load variations in key muscle groups (Erector Spinae, Rectus Abdominis, and Trapezius) and major joints (hip and knee) across posture angles.

(consistent with the manuscript’s IoMT outlook) As the backrest angle decreases below 60°, the increasing contribution of the backrest significantly reduces Erector Spinae loading. The Rectus Abdominis shows a transient load increase between ~30° and 0°, likely due to core stabilization during the transition into a supine position. The clavicular and scapular portions of the Trapezius demonstrate increased loading in the 60–30° range, highlighting the role of shoulder stabilization in posture control throughout this phase.

Joint reaction force analysis further reveals that the hip joint reaches a peak at approximately 0.5 s (around the 60° posture), while the knee joint also exhibits peak loading within the 30–60° range, indicating elevated lower-limb support and stabilization demands. Appropriately tuned device support can attenuate these joint forces, particularly at critical transition points.

Design implications. Guided by these findings, we propose four optimization strategies:

(1) define a backrest range of 0–90° to alleviate high-angle spinal muscle loads and improve sit-to-stand support;
(2) reposition and make armrests adjustable to enhance upper-limb support in the 30–60° range;
(3) optimize leg-support angles to 0–45° to balance lower-limb loading and reduce knee/hip stress;
(4) implement damping/velocity profiling to smooth speed variations during the lying process, minimizing sudden load shifts.

In summary, this data-driven structural optimization improves ergonomic performance and adaptability, meeting rehabilitation-assistance needs across diverse body types while reducing muscle fatigue and joint stress during prolonged use.

### 4.3. Limitations

First, our qualitative demand mining data is derived from JD.com reviews. Although large-scale and informative for general consumers, these data may under-represent the perspectives of clinicians, professional caregivers, and patients with complex motor disorders; hence external validity to specific clinical sub-populations is limited. Consistent with this, we already acknowledge in the manuscript that such consumer-centric sentiment may not fully reflect clinical needs, suggesting targeted validation in specialized cohorts.

Second, our biomechanical model uses the GENERICBODYMODEL template in AnyBody, which does not currently parameterize BMI or sex-specific anthropometry. This abstraction may bias predicted muscle loads and joint reactions for certain body types and stages of recovery; subsequent work should integrate individualized models to improve fidelity.

Finally, while we provide a structured comparison to IEC requirements and commercial devices, broader clinical usability (e.g., workflow fit, training burden) and long-term durability under real-world use were not tested in this study and warrant prospective investigation.

### 4.4. Directions for Future Research

(1) Clinical and multi-scenario validation. Combine wearable sensing with longitudinal trials across hospitals, rehabilitation centers, and home settings to evaluate outcomes (comfort, fatigue, adherence) and to calibrate assistance policies over time.
(2) Personalized biomechanical modeling. Extend the simulation pipeline with BMI/sex-adjusted musculoskeletal models and pathological variants (e.g., hemiparesis), enabling individualized parameter recommendations.
(3) IoMT-enabled adaptive services. Operationalize interoperability for real-time monitoring and data-driven optimization updates (e.g., remote prescription of postural trajectories), while addressing privacy and security constraints (consistent with the manuscript’s IoMT outlook).
(4) Standards and benchmarking. Expand conformance testing beyond the current IEC checklist to stress scenarios and comparative trials against next-generation commercial beds, grounding claims in user-centric metrics and safety envelopes.

### 4.5. Practical Implications and Sustainability (GreenAI)

Methodologically, the simulation-first workflow reduces repeated physical prototyping, saving materials, labor, and time—thus lowering environmental and economic costs across the product life cycle. This aligns with the project’s GreenAI stance and sustainable development principles and should be maintained as a core engineering practice in subsequent iterations.

## 5. Conclusions

This study achieved coordinated optimization of support structure, muscle load distribution, joint loading patterns, and motion parameters for the back-support lifting nursing bed while maintaining its fundamental assistive functions. The integration of emotional design principles with biomechanical simulation established a human-centered, data-driven optimization approach (single design cycle with simulation verification) from user demand identification to structural optimization and simulation verification. Accordingly, we avoid claiming a fully closed loop in this work. The results validated the effectiveness of the proposed design in improving comfort, safety, and adaptability, offering a novel perspective for the intelligent and personalized development of rehabilitation nursing equipment.

A numerical summary of the article’s achievements is as follows:

1. Data-driven need mining. We assembled 11,132 valid user reviews and constructed a behavior–emotion dual-channel demand map, yielding 5 thematic clusters via Word2Vec + K-Means (see Table 3).
2. Fuzzy Kano prioritization. Across 35 service attributes (see Table 10), the fuzzy Kano analysis identified 31 priority requirements in total—12 One-dimensional, 11 Must-be, and 8 Attractive—with 4 Indifferent and 0 Reverse/Questionable items (see Table 8, Table 9 and Table 10).
3. Biomechanical verification. AnyBody simulations covered a 90° → 0° backrest sweep. The hip joint reaction peaked at ≈0.5 s (around 60°), while the knee joint peaked within 30–60°. Erector Spinae loading was maximal at 90° (notably T9–L1) and decreased markedly <60°; Rectus Abdominis rose transiently from ≈30° → 0°; Trapezius (clavicular/scapular parts) increased within 60–30° (see Figure 7, Figure 8, Figure 9 and Figure 10 for muscles; Figure 11, Figure 12, Figure 13 and Figure 14 for joint reactions).
4. Design outcomes. Guided by these findings, we propose four implementable strategies: (1) define a 0–90° backrest range; (2) reposition and make armrests adjustable to support 30–60°; (3) set leg-support angles to 0–45°; and (4) apply damping/velocity profiling to smooth transitions.
5. Integration and compliance. The optimized architecture is IoMT-ready and was benchmarked against relevant ISO/IEC medical-bed requirements and existing commercial beds across four dimensions: functionality, safety, comfort, and adjustability (see relevant sections in the manuscript).

Beyond its functional capabilities, the design methodology adopted for this project emphasizes sustainability through a reduction in waste in the product development process. By minimizing the need for multiple physical prototypes and optimizing the use of materials and labor, the design process not only reduces environmental impact but also ensures more efficient resource use. This approach to design, grounded in sustainable development principles, offers significant long-term benefits, both in terms of product performance and environmental responsibility. As the technology evolves, further integration of sustainable practices can be expected, positioning this product as a leader in eco-friendly healthcare solutions.

Future research will incorporate wearable sensor data and clinical trials to evaluate applicability across diverse user populations and rehabilitation stages and explore AI-based adaptive control and personalized parameter recommendations to enable widespread use of the back-support lifting nursing bed in multi-scenario, long-term rehabilitation contexts.

## Figures and Tables

**Table 1:** TF-IDF keyword frequency statistics.

IDX	Words	TF-IDF	Occurrences
1	Good	0.053514	2897
2	Quality	0.044158	2361
3	Elder	0.037622	296
4	Satisfy	0.029626	1390
5	Receive	0.023856	1186
6	Logistics	0.023491	12,259
7	Thing	0.022490	974
8	Quick	0.021937	1084
9	Install	0.020953	1188
10	Like	0.020541	904
11	Worth	0.020221	897
12	Effect	0.018578	794
13	Service	0.018461	1099
14	Purchase	0.018458	832
15	Fixed use	0.017796	515

TF-IDF represents the importance weight of the keyword; Number indicates the frequency of occurrence of the keyword in the review text. Occurrences is the total frequency of the word in the whole corpus, which can exceed the number of comments.

**Table 2:** Results of the LDA topic model.

Topic	Classes	Keywords
Topic 1	Overall usage experience	Good, effect, favorable comments, feeling, useful
Topic 2	Installation and Guidance Service	Installation, customer service, guidance, mode, first time
Topic 3	Product quality and price	Quality, price, cheap, recommendation, affordability
Topic 4	Material and design details	Workmanship, material quality, design, cost-effectiveness, comprehensive functionality
Topic 5	Rehabilitation function and applicability	Recovery, elderly people, exercise, function, suitable
Topic 6	Logistics and Packaging Satisfaction	Logistics, delivery, packaging, speed, satisfaction
Topic 7	Applicability to specific groups of people	Old person, care, bed, patient, turn over
Topic 8	After-sales service and trust factor	Deserving, customer service, trust, service, recommendation

Classes represent the general description of the topic categories; Keywords indicate the high-frequency keywords under the corresponding categories.

**Table 3:** Results of the topic model based on Word2Vec and K-Means clustering.

Cluster	Classes	Keywords
Cluster 1	Applicability to Specific User Groups	Old people, rehabilitation, care, function, quality
Cluster 2	Logistics and Distribution	Logistics, quality, customer service, satisfaction, express delivery
Cluster 3	Price Evaluation	Satisfaction, delivery, price, service, value for money
Cluster 4	User Experience	WUOld person, function, rehabilitation, practical, bed
Cluster 5	Intention to Recommend	Quality, recommendation, satisfaction, usability, logistics

Classes represent the general description of the clustering categories; Keywords indicate the high-frequency keywords under the corresponding categories. Note: Class labels were standardized for clarity and corrected for translation/typos.

**Table 4:** Classification of user requirements.

Primary Indicator	Secondary Indicator	Third-Level Indicators	IDX	Feature
Security	Adjustability	Adjusting resistance	A1	A
		Multi-level actuation rate (backrest/leg rest)	A2	M
		Range of activity	A3	O
	Stability of structures	Prevent overstretching	A4	O
		Prevent sprains	A5	O
	Antiskid		A6	M
	Joint protection		A7	A
	Emergency shutdown device		A8	M
Human factors	Height adjustable		B1	O
	Weight regulation		B2	M
	Full joint range of motion		B3	O
	Adjustable armrests		B4	A
User-friendly interaction	Large-sized buttons		C1	M
	Emotionalized design	Friendly design	C2	A
		Encouraging feedback	C3	O
Product appearance design	Portability/Mobility	Folded structure	D1	M
		Wheeled base	D2	M
	Light weight	Sensor hidden	D3	O
		Cable concealment	D4	O
	Material and Appearance	Soft colors	D5	I
		Simple and fashionable appearance	D6	A
		Home-style color scheme	D7	I
	Economy and Sustainability	Normalizer	D8	O
		Easy to install	D9	M
Applicability	Big font		E1	M
	High contrast		E2	O
	Multimodal system	Language	E3	O
		Voice	E4	M
		Shake	E5	A
	Low motor coordination ability		E6	I
Serviceability and Maintenance	Operational usability (controls, modes)		F1	O
	Price		F2	A
	Manner		F3	I
	Maintenance turnaround and spare-part availability		F4	A
	Simple installation		F5	M

Primary indicator represents the first-level dimension of user requirements; Secondary indicator provides a more detailed subdivision; Third-level indicators describe specific measurable features; IDX corresponds to the index code; Feature indicates the Kano attribute classification (A: Attractive, O: One-dimensional, M: Must-be, I: Indifferent). “Speed/rate” in this table denotes the device actuation rate of the backrest/leg-rest mechanisms; it must not be interpreted as logistics or delivery speed.

**Table 5:** Questionnaire for the fuzzy Kano model.

Service Elements	Very Satisfied	As It Should Be	Indifferent	Reluctantly Acceptable	Dissatisfied
Available	0.6	0.4	0	0	0
Not available	0	0	0.1	0.1	0.8

Service elements indicate the attributes being evaluated; the values under each option (Very satisfied, As it should be, Indifferent, Reluctantly acceptable, Dissatisfied) represent the distributed weights assigned by respondents, with the sum constrained to 1.

**Table 6:** Example of questionnaire information.

		Very Satisfied	As It Should Be	Indifferent	Reluctantly Acceptable	Dissatisfied
Adjusting resistance	Feature Present	0.6	0.4	0	0	0
	Feature Absent	0	0	0	0.2	0.8

Feature Present and Feature Absent indicate the availability of the evaluated attribute; the values under each option (Very satisfied, As it should be, Indifferent, Reluctantly acceptable, Dissatisfied) represent the distributed weights assigned by respondents, with the sum constrained to 1.

**Table 7:** Demand attribute classification and evaluation based on the fuzzy Kano model.

		Feature Absent				
		Dissatisfied	Reluctantly Accept	Dissatisfied	Must-Be/As Expected	Very Satisfied
Feature Present	Dissatisfied	Q	R	R	R	R
	Reluctantly Accept	M	I	I	I	R
	Indifferent	M	I	I	I	R
	Must-be/As Expected	M	I	I	I	R
	Very Satisfied	O	A	A	A	Q

Reverse-type requirements (R), Questionable requirements (Q), One-dimensional requirements (O), Must-be requirements (M), Attractive requirements (A), and Indifferent requirements (I).

**Table 8:** Analysis results of the fuzzy Kano model.

	IDX	Service Content	O	M	I	R	A	Q	Result	Sign
Security	A1	Adjusting resistance	94	14	41	3	117	0	Attractive	A
	A2	Multi-level actuation rate (backrest/leg rest)	79	99	43	11	31	6	Must-be	M
	A3	Range of activity	175	20	43	26	2	3	One-dimensional	O
	A4	Prevent overstretching	184	7	47	28	0	3	One-dimensional	O
	A5	Prevent sprains	142	30	46	11	40	0	One-dimensional	O
	A6	Antiskid	89	108	55	0	17	0	Must-be	M
	A7	Joint protection	90	41	41	3	94	0	Attractive	A
	A8	Emergency shutdown device	95	101	35	23	12	3	Must-be	M
Human-engineering	B1	Height adjustable	120	26	49	7	63	4	One-dimensional	O
	B2	Weight regulation	81	110	59	0	19	0	Must-be	M
	B3	Full joint range of motion	137	38	35	18	38	3	One-dimensional	O
	B4	Adjustable armrests	104	18	32	3	112	0	Attractive	A
User-friendly interaction	C1	Large-sized buttons	83	137	34	0	15	0	Must-be	M
	C2	Friendly design	97	14	41	3	114	0	Attractive	A
	C3	Encouraging feedback	137	34	46	12	40	0	One-dimensional	O
Product appearance design	D1	Folded structure	77	104	41	12	29	6	Must-be	M
	D2	Wheeled base	72	113	39	9	32	4	Must-be	M
	D3	Sensor hidden	84	54	48	12	71	0	One-dimensional	O
	D4	Cable concealment	105	48	57	6	53	0	One-dimensional	O
	D5	Soft colors	48	30	94	9	88	0	Indifference	I
	D6	Simple and fashionable appearance	85	36	47	0	101	0	Attractive	A
	D7	Home-style color scheme	51	28	92	9	89	0	Indifference	I
	D8	Normalizer	80	47	53	12	77	0	One-dimensional	O
	D9	Easy to install	94	97	37	20	19	2	Must-be	M
Applicability	E1	Big font	92	102	56	0	19	0	Must-be	M
	E2	High contrast	134	53	27	20	31	4	One-dimensional	O
	E3	Language	129	60	29	4	47	0	One-dimensional	O
	E4	Voice	86	93	37	12	35	6	Must-be	M
	E5	Shake	73	42	29	0	125	0	Attractive	A
	E6	Low motor coordination ability	48	21	99	6	95	0	Indifference	I
Serviceability and Maintenance	F1	Operational usability (controls, modes)	99	50	47	3	70	0	One-dimensional	O
	F2	Price	70	43	42	3	111	0	Attractive	A
	F3	Manner	53	25	95	6	90	0	Indifference	I
	F4	Maintenance turnaround and spare-part availability	68	49	40	5	107	0	Attractive	A
	F5	Simple installation	96	104	54	0	15	0	Must-be	M

O = One-dimensional, M = Must-be, I = Indifferent, R = Reverse, A = Attractive, Q = Questionable. The “Result” column represents the final Kano category classification, and “Sign” indicates the corresponding symbol used in subsequent analysis.

**Table 9:** Summary of demand attribute classification.

Feature	IDX
O (Retain)	A3, A4, A5, B1, B3, C3, D3, D4, D8, E2, E3, F1
M (Retain)	A2, A6, A8, B2, C1, D1, D2, D9, E1, E4, F5
A (Retain)	A1, A7, B4, C2, D6, E5, F2, F4
I (Retain)	D5, D7, E6, F3
R (Remove)	
Q (Remove)	

O = One-dimensional, M = Must-be, A = Attractive, I = Indifferent, R = Reverse, Q = Questionable. “Retain” indicates attributes that are kept for design consideration, while “Remove” indicates attributes excluded from further analysis.

**Table 10:** Customer satisfaction coefficients for 35 service attributes.

Content	Sign	Better	Worse	Content	Sign	Better	Worse
Adjusting resistance	A	0.793	−0.406	Cable concealment	O	0.601	−0.582
Multi-level actuation rate (backrest/leg rest)	M	0.437	−0.706	Soft colors	I	0.523	−0.300
Range of activity	O	0.738	−0.813	Simple and fashionable appearance	A	0.691	−0.450
Prevent overstretching	O	0.773	−0.803	Home-style color scheme	I	0.538	−0.304
Prevent sprains	O	0.705	−0.667	Normalizer	O	0.611	−0.494
Antiskid	M	0.394	−0.732	Easy to install	M	0.457	−0.773
Joint protection	A	0.692	−0.492	Big font	M	0.413	−0.721
Emergency shutdown device	M	0.440	−0.807	High contrast	O	0.673	−0.763
Height adjustable	O	0.709	−0.566	Language	O	0.664	−0.713
Weight regulation	M	0.372	−0.710	Voice	M	0.482	−0.713
Full joint range of motion	O	0.706	−0.706	Shake	A	0.736	−0.428
Adjustable armrests	A	0.812	−0.459	Low motor coordination ability	I	0.544	−0.262
Large-sized buttons	M	0.364	−0.818	Operational usability (controls, modes)	O	0.635	−0.560
Friendly design	A	0.793	−0.417	Price	A	0.680	−0.425
Encouraging feedback	O	0.689	−0.665	Manner	I	0.544	−0.297
Folded structure	M	0.422	−0.721	Maintenance turnaround and spare-part availability	A	0.663	−0.443
Wheeled base	M	0.406	−0.723	Simple installation	M	0.413	−0.743
Sensor hidden	O	0.603	−0.537				

“Better” and “Worse” represent the customer satisfaction and dissatisfaction coefficients calculated for each service attribute. “Sign” corresponds to the Kano category classification, where A = Attractive, O = One-dimensional, M = Must-be, I = Indifferent.

## Data Availability

Data are available on request from the corresponding author.
